# Supplementary material for: Computational Analysis of Exotic Molecular and Atomic Vibrations in Ice XV
Source: Molecules. 2019 Aug 27;24(17):3115. doi: 10.3390/molecules24173115 (PMC6749349; doi:10.3390/molecules24173115)
Supplement: Supplementary file 1 [file molecules-24-03115-s001.zip › S1-5/S1.docx]

| **Normal Modes**  **(1.4GPa)** | **Normal Modes**  **(0.9GPa)** | **Raman Exp. [8/11]** |
| --- | --- | --- |
| 66 | 73 |  |
| 80 | 106 |  |
| 82 | 107 |  |
| **96** | **136** | **114/113** |
| 104 | 138 |  |
| 112 | 148 |  |
| 115 | 150 |  |
| 123 | 158 |  |
| 128 | 162 |  |
| 143 | 165 |  |
| 143 | 167 |  |
| **147** | **175** | **158/159** |
| 151 | 181 |  |
| 154 | 182 |  |
| 159 | 184 |  |
| 162 | 185 |  |
| 167 | 192 |  |
| **180** | **201** | **186/185** |
| 181 | 206 |  |
| **207** | **221** | **220/215** |
| 214 | 225 |  |
| 218 | 231 |  |
| **221** | **232** | **230/** |
| 222 | 234 |  |
| **223** | **236** | **300/** |
| 260 | 275 |  |
| **270** | **285** | **312/303** |
| **486** | **477** | **451/450** |
| 500 | 484 |  |
| 529 | 508 |  |
| **532** | **531** | **499/495** |
| 558 | 550 |  |
| **561** | **566** | **548/544** |
| 571 | 579 |  |
| 604 | 611 |  |
| 610 | 618 |  |
| 620 | 621 |  |
| 626 | 631 |  |
| 640 | 635 |  |
| 644 | 649 |  |
| 650 | 652 |  |
| 665 | 662 |  |
| 674 | 671 |  |
| 680 | 677 |  |
| 705 | 685 |  |
| 706 | 702 |  |
| 724 | 718 |  |
| 726 | 731 |  |
| 761 | 748 |  |
| 806 | 775 |  |
| 831 | 817 |  |
| 867 | 864 |  |
| 878 | 867 |  |
| 898 | 892 |  |
| 908 | 913 |  |
| 932 | 928 |  |
| 943 | 936 |  |
| 1608 | 1609 |  |
| 1629 | 1629 |  |
| 1648 | 1648 |  |
| 1657 | 1655 |  |
| 1659 | 1656 |  |
| 1666 | 1660 |  |
| 1692 | 1684 |  |
| 1697 | 1692 |  |
| 1708 | 1705 |  |
| 1712 | 1709 |  |
| 3284 | 3278 |  |
| **3286** | **3280** | **3224/3222** |
| 3335 | 3325 |  |
| **3337** | **3326** | **3335/** |
| 3352 | 3339 |  |
| **3352** | **3341** | **3345/3349** |
| 3365 | 3354 |  |
| 3367 | 3357 |  |
| 3373 | 3366 |  |
| 3375 | 3367 |  |
| 3404 | 3402 |  |
| **3405** | **3403** | **3396/** |
| 3428 | 3429 |  |
| **3441** | **3432** | **3425/3420** |
| 3451 | 3442 |  |
| 3454 | 3448 |  |
| 3478 | 3452 |  |
| 3479 | 3461 |  |
| 3496 | 3472 |  |
| 3504 | 3485 |  |
